# Supplementary material for: Magnetoelectric coupling in multiferroics probed by optical second harmonic generation
Source: Nat Commun. 2023 Apr 20;14:2274. doi: 10.1038/s41467-023-38055-x (PMC10119081; doi:10.1038/s41467-023-38055-x)
Supplement: Supplementary file 1 — Supplementary Information [file 41467_2023_38055_MOESM1_ESM.pdf]

SUPPLEMENTARY INFORMATION for

Magnetoelectric Coupling in Multiferroics Probed by Optical Second Harmonic Generation

Shuai Xu[1,2,†], Jiesu Wang[4,†], Pan Chen[1,†], Kuijuan Jin[1,2,3,*], Cheng Ma[1,2], Shiyao Wu[4], Er-Jia Guo[1,2], Chen Ge[1,2], Can Wang[1,2,3], Xiulai Xu[1,5], Hongbao Yao[1,2], Jingyi Wang[1], Donggang Xie[1], Xinyan Wang[1,2], Kai Chang[4], Xuedong Bai[1,2,3], Guozhen Yang[1,2]

[1] Beijing National Laboratory for Condensed Matter Physics, Institute of Physics, Chinese Academy of Sciences, Beijing 100190, China
[2] University of Chinese Academy of Sciences, Beijing 100049, China
[3] Songshan Lake Materials Laboratory, Dongguan, Guangdong 523808, China
[4] Beijing Academy of Quantum Information Sciences, Beijing 100193, China
[5]State Key Laboratory for Mesoscopic Physics and Frontiers Science Center for Nano-optoelectronics, School of Physics, Peking University, Beijing 100871, China
[†] These authors contributed equally: Shuai Xu, Jiesu Wang and Pan Chen
[*]Author to whom correspondence should be addressed: kjjin@iphy.ac.cn

CONTENTS

1. Characterization of basic properties for BFO films
2. HAADF-STEM images and atomically resolved energy-dispersive X-ray spectroscopy (EDS) mapping of the BFO/SAO/STO
3. Atomic-scale HAADF-STEM images and fast fourier transform (FFT) patterns of the BFO/STO films
4. SHG measurements at different incident laser powers
5. RA-SHG measurements of BFO/STO films under different temperatures without magnetic field
6. Temperature-dependent SHG signal contributed by ferroelectric order of BFO/STO films extracted from RA-SHG results at different temperatures
7. Room temperature RA-SHG measurements of freestanding BFO films under different magnetic fields
8. First-principles calculation results of 2×2×2 BFO supercell under different magnetic structures
9. Schematic diagram for the possible mechanisms of magnetism enhancement in BFO films
10. M-H results of about 53-nm-thick epitaxial and freestanding BFO films
11. Magnetic field-dependent SHG signal contributed by antiferromagnetic order and ferroelectric order of freestanding BFO films extracted from RA-SHG results at different magnetic fields
12. Mathematical expressions for SHG processes

1. Characterization of basic properties for BFO films

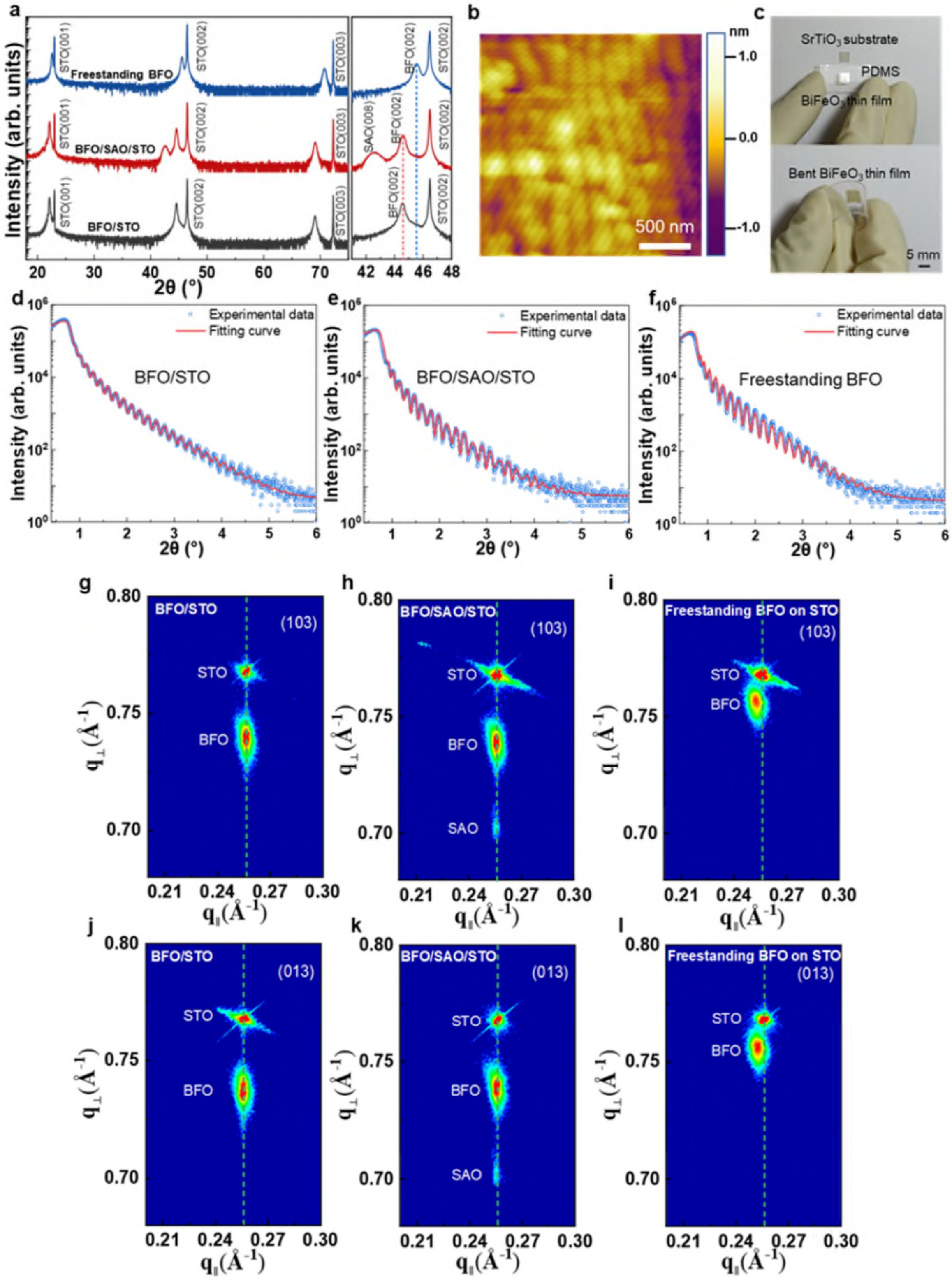

Supplementary Figure 1 | a X-ray diffraction patterns of BFO/STO, BFO/SAO/STO, and freestanding BFO films transferred onto STO. The red and blue dashed lines indicate the shift of

the (002) diffraction peak of freestanding BFO films. **b** Surface morphology of transferred freestanding BFO onto 0.7 *wt*% Nb-doped $SrTiO_3$ (001) substrate by atomic force microscopy. **c** A 5 mm×5 mm freestanding BFO film transferred on the PDMS exhibiting good flexibility. **d-f** X-ray reflectivity measurements of BFO/STO, BFO/SAO/STO, and freestanding BFO films, respectively. The solid lines are the bests fit to the experimental data (open symbols), with calculated thickness of the BFO film in **d** about 47.0 nm, thicknesses of BFO and SAO in **e** about 46.5 nm and 16.7 nm respectively and the thickness of the freestanding BFO in **f** about 45.6 nm. It's worth noting that the thickness of all the BFO layers among these samples are almost the same. **g-i** RSM of BFO/STO, BFO/SAO/STO, and freestanding BFO films around (103) peak, respectively. **j-l** RSM of BFO/STO, BFO/SAO/STO, and freestanding BFO films around (013) peak, respectively. The results show that the BFO/STO and BFO/SAO/STO films are both co-epitaxially grown.

2. HAADF-STEM images and atomically resolved energy-dispersive X-ray spectroscopy (EDS) mapping of the BFO/SAO/STO

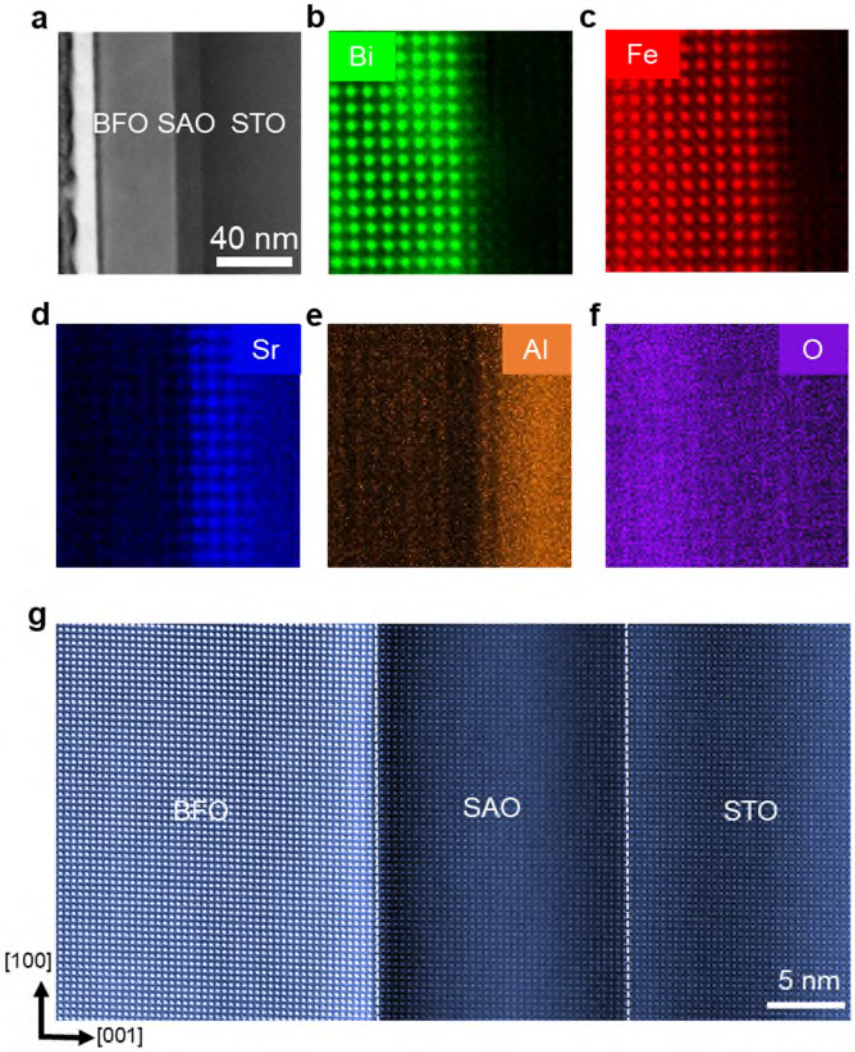

Supplementary Figure 2 | a A low-magnification scanning TEM high-angle annular dark-field (STEM-HAADF) image of the BFO/SAO/STO, where the bright area indicates the BFO layer and the dark area indicates SAO buffer layer grown on STO substrate. **b-f** Atomically resolved energy-dispersive X-ray spectroscopy (EDS) mapping of the Bi, Fe, Sr, Al, and O elements. The results show the good quality of the BFO films without any dislocation. **g** HAADF-STEM images of the BFO/SAO/STO heterostructures. The interfaces between the BFO layer and SAO layer, as well as that one between SAO layer and STO substrate can be clearly distinguished from the HAADF-STEM images.

3. Atomic-scale HAADF-STEM images and fast fourier transform (FFT) patterns of the BFO/STO films

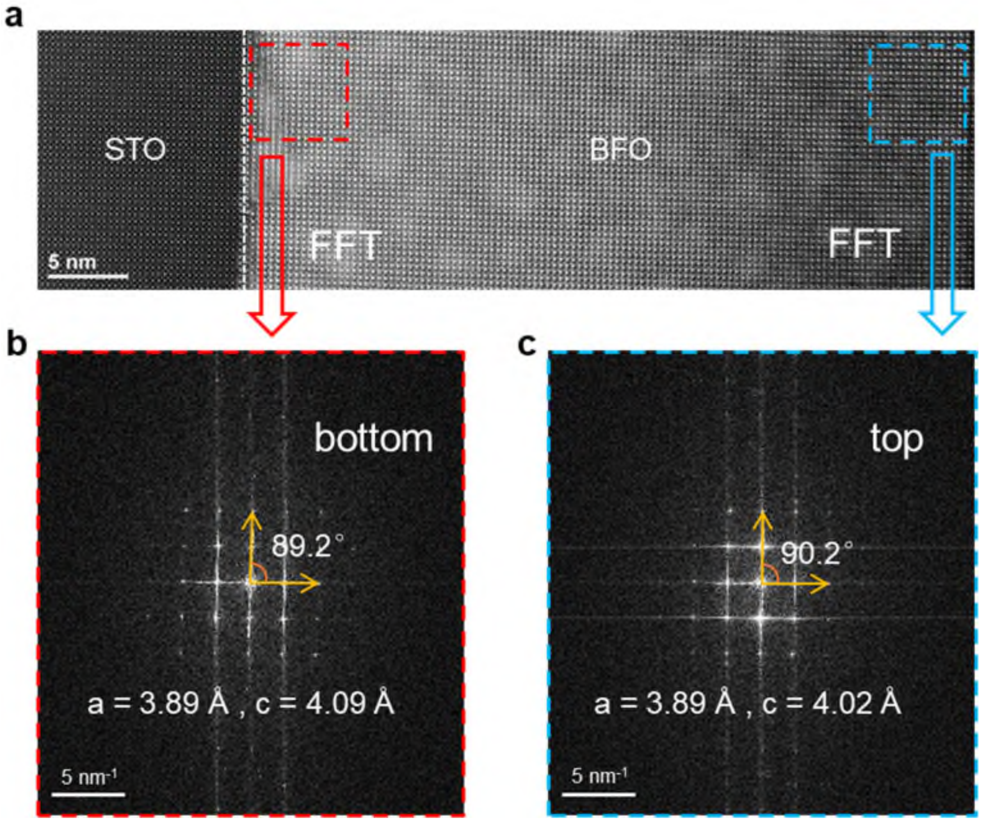

Supplementary Figure 3 | a Atomic-scale HAADF-STEM images of the BFO/STO films. **b** and **c** Fast fourier transform (FFT) patterns of the bottom BFO and the top BFO, respectively.

4. SHG measurements at different incident laser powers

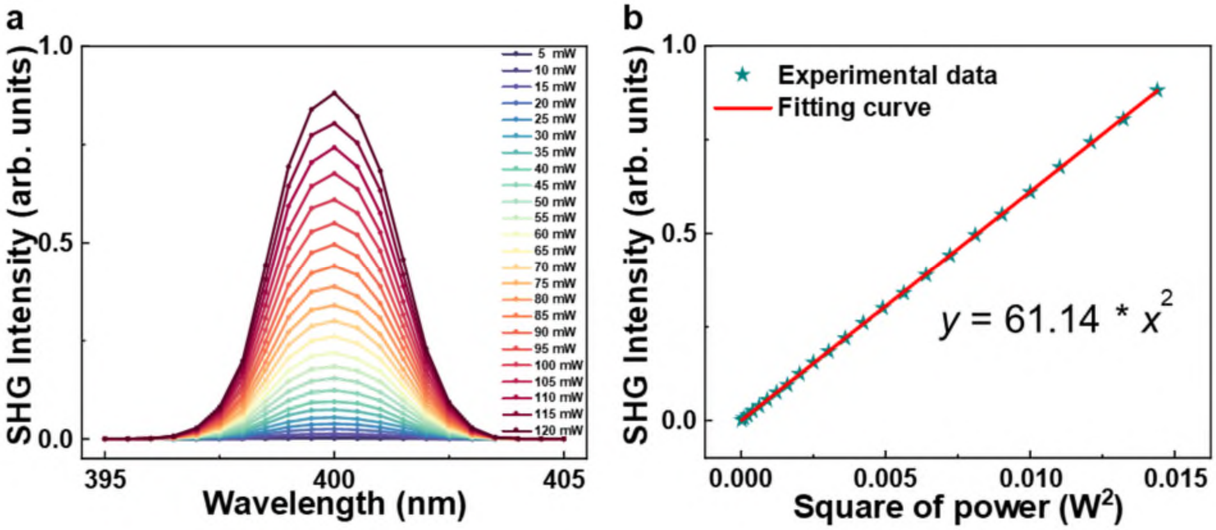

Supplementary Figure 4 | a Incident light power dependent SHG spectra of BFO/STO. **b** Linear relationship between the intensity on 400 nm of **a** and the square of incident light power, confirming the SHG response. For other samples, they exhibit the similar properties.

5. RA-SHG measurements of BFO/STO films under different temperatures without magnetic field

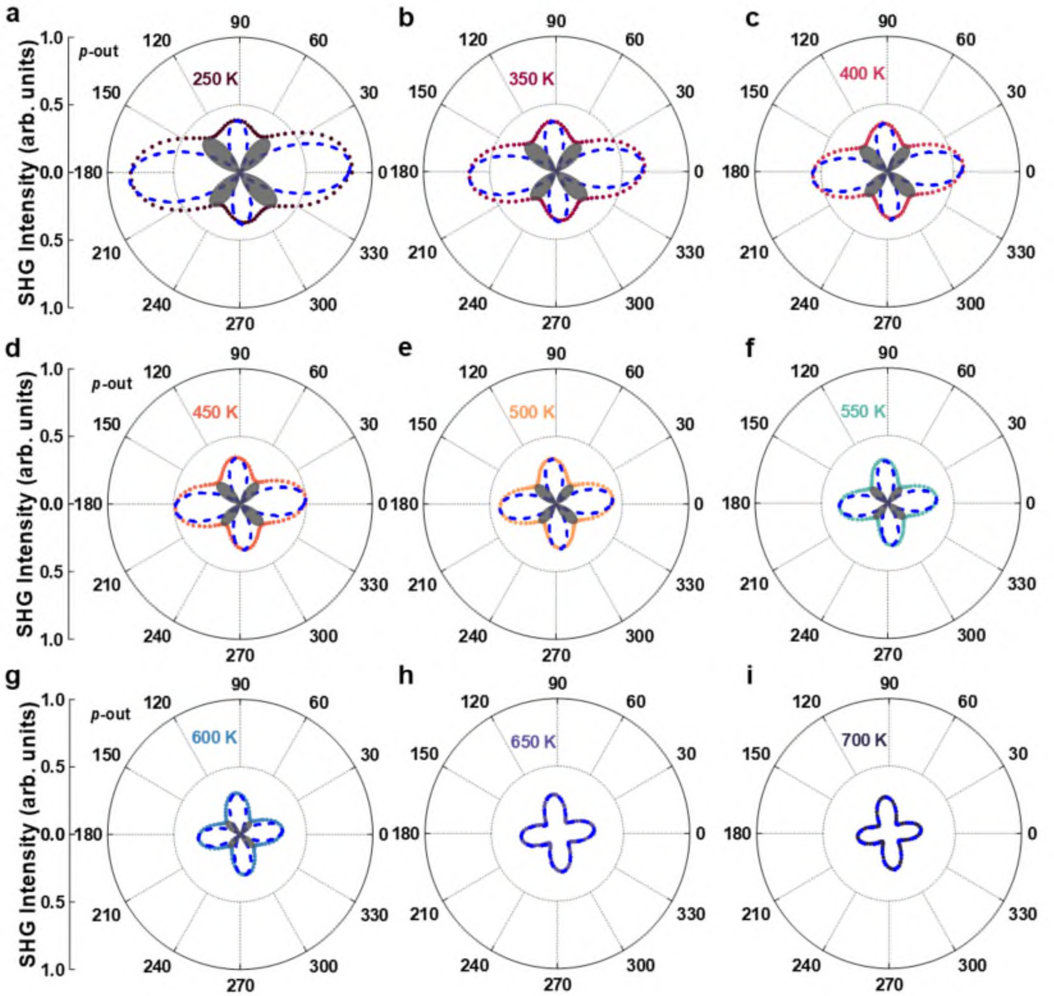

Supplementary Figure 5 | a-g RA-SHG measurements of BFO/STO films below the Néel temperature (about 618 K), both the antiferromagnetic order and the ferroelectric order contribute to the SHG signal, and their coupling leads to the asymmetry of the RA-SHG patterns. **h-i** RA-SHG patterns of BFO/STO films above Néel temperature, in which the antiferromagnetic order disappears and only the ferroelectric order contributes to the SHG signal. With temperature increasing, both the antiferromagnetic and ferroelectric orders are suppressed. When the temperature is above the Néel temperature, the sample transform from the antiferromagnetic phase to the paramagnetic phase, leaving only the ferroelectric order. The scatters are the experimental data, the bule dashed lines represent the contribution of the ferroelectric order to the SHG signal, and the gray shadows represent the contribution of the antiferromagnetic order to the SHG signal.

6. Temperature-dependent SHG signal contributed by ferroelectric order of BFO/STO films extracted from RA-SHG results at different temperatures

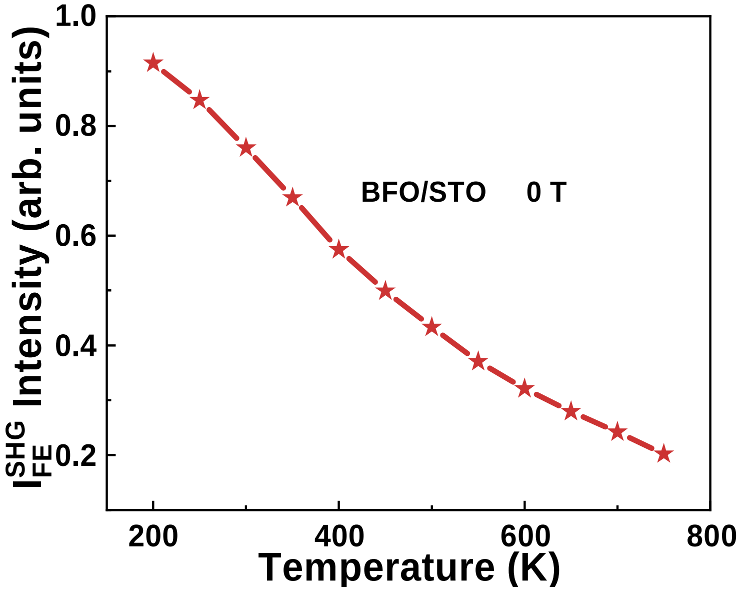

Supplementary Figure 6 | Because the Curie temperature ($T_C \approx 1100$ K) of BFO is relatively high, in the range of 200 K to 750 K, the polarity of BFO will become weak as the temperature increases but will not disappear. Therefore, the SHG intensity of BFO will gradually decrease with the increase of temperature, but it will not drop to zero.

7. Room temperature RA-SHG measurements of freestanding BFO films under different magnetic fields

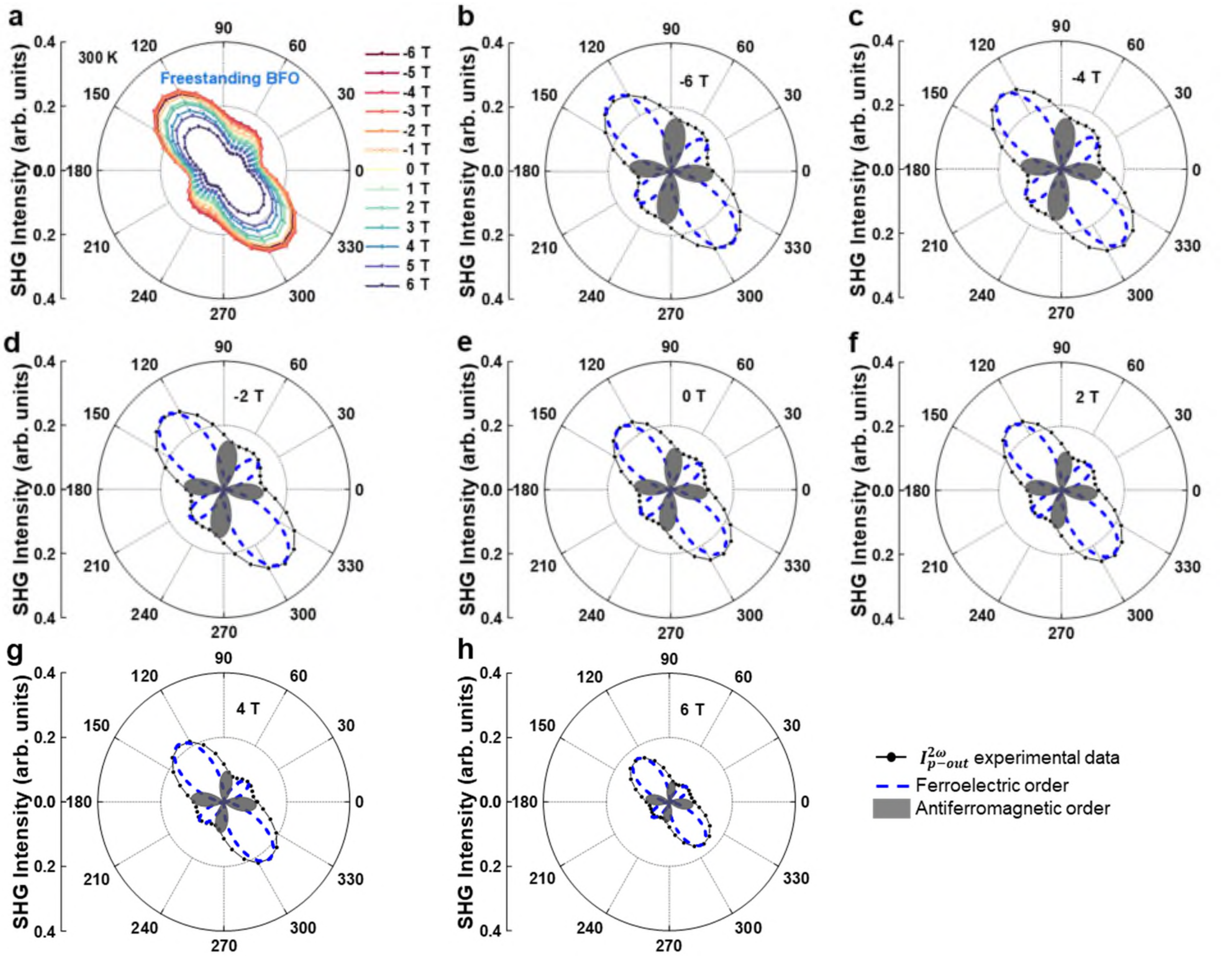

Supplementary Figure 7 | a RA-SHG of freestanding BFO films under different magnetic fields at room temperature. **b-h** Theoretical fitting results of -6 T, -4 T, -2 T, 0 T, 2 T, 4 T, and 6 T RA-SHG patterns respectively. The dotted lines are the experimental data, and the bule dashed lines represent the contribution of the ferroelectric order to the SHG signal. The gray shadows represent the contribution of the antiferromagnetic order to the SHG signal. It can be seen that both parts vary significantly with the magnetic field.

8. First-principles calculation results of $2\times2\times2$ BFO supercell under different magnetic structures

As shown in Fig. S8, we have calculated the spontaneous polarization of BFO with intrinsic FM order and G-type AFM order, respectively. The lattice constants were fixed as our XRD and TEM experimental results of the STO-BFO during our first-principles calculations (a=b=3.89 Å, c=4.06 Å). From Table S1 and Fig. S8, we can see that the electronic dipole and ionic dipole are both larger in BFO with G-type antiferromagnetic order than those with the ferromagnetic order in all three directions, namely *a* ([100]), *b* ([010]), and *c* ([001]). The enlargement of polarization demonstrates theoretically that the antiferromagnetic order would enhance the polarization.

Supplementary Table 1: Ionic and electronic polarization of BFO (a=b=3.89 Å, c=4.06 Å) in the directions *a*, *b*, and *c*, respectively.

	$P_{ion.,a}$ $\|e\|\cdot\dot{A}$	$P_{ion.,b}$ $\|e\|\cdot\dot{A}$	$P_{ion.,c}$ $\|e\|\cdot\dot{A}$	$P_{ele.,a}$ $\|e\|\cdot\dot{A}$	$P_{ele.,b}$ $\|e\|\cdot\dot{A}$	$P_{ele.,c}$ $\|e\|\cdot\dot{A}$
G-AFM	-149.3	-149.3	-155.7	0.547	0.547	-2.681
FM	-149.2	-149.2	-155.4	0.443	0.443	-2.329

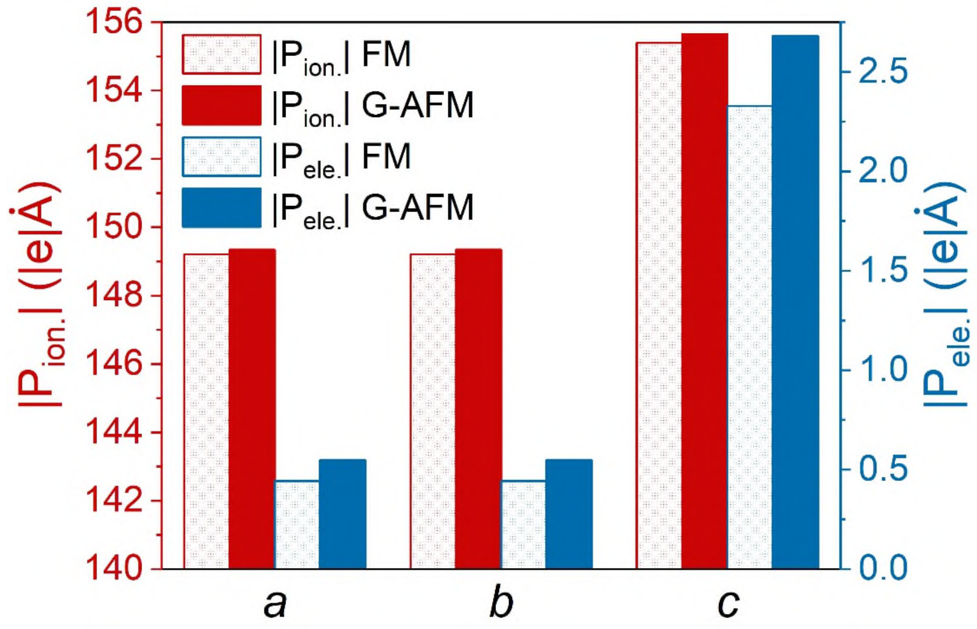

Supplementary Figure 8 | First-principles calculation results of 2 × 2 × 2 BFO supercell under different magnetic structures. The absolute value of the electronic dipole moment (in blue) and the ionic dipole moment (in red) of BFO with ferromagnetic (FM) order (hollow) and G-type antiferromagnetic (G-AFM) order (filled) in directions a, b, and c.

9. Schematic diagram for the possible mechanisms of magnetism enhancement in BFO films

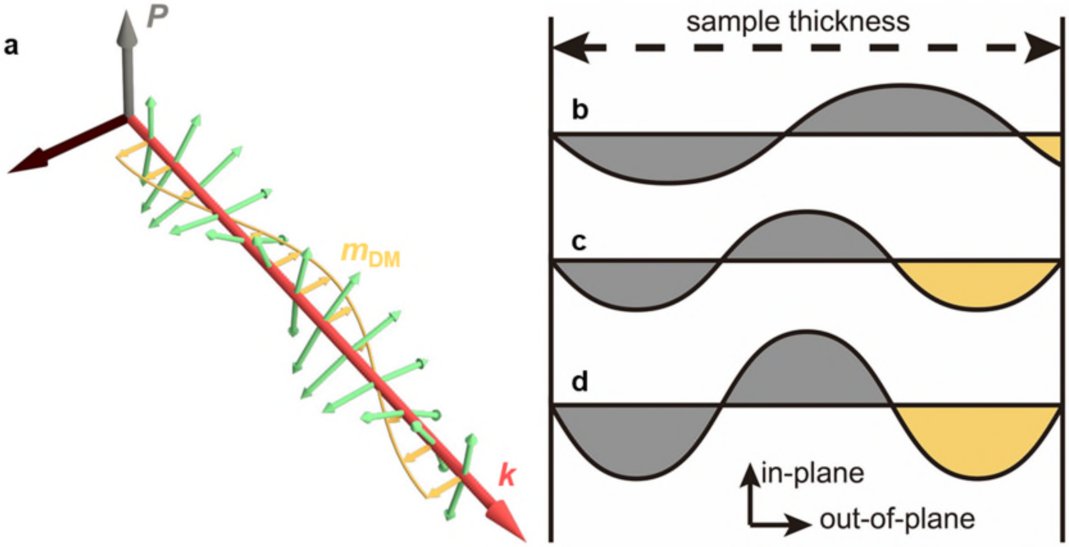

Supplementary Figure 9 | a Schematic diagram of the cycloidal local magnetic moment (orange arrows) induced by the antiferromagnetic cycloidal order. **b** and **c** The residual magnetic moment (orange shadow) enlargement resulted by the variation of the period of the antiferromagnetic cycloidal order[1, 2]. **d** The residual magnetic moment enlargement resulted by the weakening of collinearity and enhancement of m_{DM} with the transition of propagation direction k[3].

10. M-H results of about 53-nm-thick epitaxial and freestanding BFO films

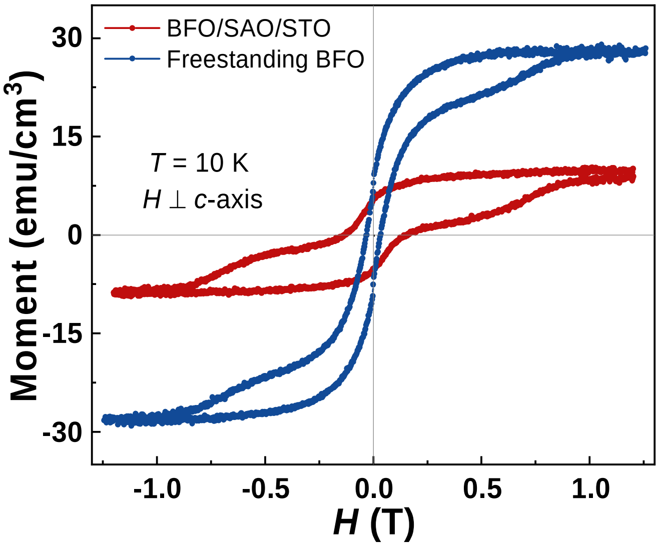

Supplementary Figure 10 | The red dotted line represents the data of about 53-nm-thick epitaxial BFO thin film, and the blue dotted line represents the data of about 53-nm-thick freestanding BFO thin film.

11. Magnetic field-dependent SHG signal contributed by antiferromagnetic order and ferroelectric order of freestanding BFO films extracted from RA-SHG results at different magnetic fields

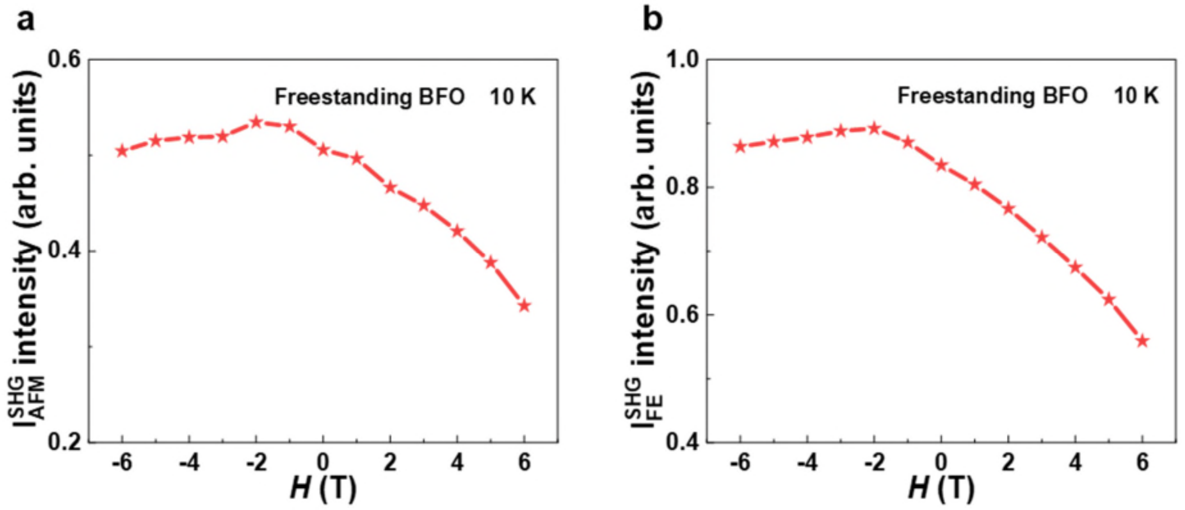

Supplementary Figure 11 | a and **b** Magnetic field-dependent SHG signal contributed by antiferromagnetic order and ferroelectric order extracted from the RA-SHG results of freestanding BFO films at different magnetic field, respectively.

12. Mathematical expressions for SHG processes

The second harmonic generation in the nonlinear optical process can be expressed as: $\boldsymbol{P}(2\omega) = \chi^{(2)} \colon \boldsymbol{E}(\omega)\boldsymbol{E}(\omega)$, where $\chi^{(2)}$ is the second-order nonlinear optical susceptibility tensor of the three-rank, and $E(\omega)$ is the incident optical electric field of the laser with a frequency ω. Under the multi-level approximation, it can be expressed as: $\chi^{(2)}(\omega, q) \approx \chi^{(2)}_{ED}(\omega) + \chi^{(3)}_{EQ}(\omega) q + o(q^2)$, where ω and q are the incident light frequency and photon wave vector, respectively [4]. In general, the three-rank electric dipole (ED) term $\chi^{(2)}_{ED}(\omega)$ contributes the most, while the electric quadrupole (EQ) term $\chi^{(3)}_{EQ}(\omega) q$ and other higher-order terms contribute less. The three-rank $\chi^{(2)}_{ED}(\omega)$ is constrained not only by the formal symmetry properties of the tensor itself, but also by the nonlinear medium symmetry. If the medium has inversion symmetry, the components in $\chi^{(2)}_{ED}(\omega)$ will follow the relationship: $\chi^{ED}_{ijk} = -\chi^{ED}_{ijk}$, resulting in $\chi^{ED}_{ijk} = 0$. Therefore, $\chi^{(2)}_{ED}(\omega)$ can only be produced in non-centrosymmetric medium (such as ferroelectric materials, surfaces, and interfaces of materials) [5, 6]. For ferroelectric crystalline materials that can undergo an ED process, from the signal obtained from the rotational anisotropy SHG measurements, information related to the crystal symmetry, such as lattice structure and crystal orientation, can be analytically extracted.

For a crystalline material with magnetic order, the $\chi^{(2)}$ can be expressed as: $\chi^{(2)}_{ED} = \varepsilon_0 \left(\chi^{(\mathrm{i})} + \chi^{(\mathrm{c})} \right)$, where the time-invariant tensor $\chi^{(\mathrm{i})}$ and the time-noninvariant tensor $\chi^{(\mathrm{c})}$ represent the crystallographic (symmetry breaking caused by ferroelectric) and magnetic orders structure (symmetry breaking caused by antiferromagnetic), respectively [7, 8]. For the multiferroic BFO material, the SHG signal contains both parts. With an approximation that considers only the electric dipole, the SHG source term $S(2\omega)$ is related to the light-induced nonlinear polarization $\boldsymbol{P}(2\omega)$ in the following way: $S(2\omega) = \mu_0 \dfrac{\partial^2 \boldsymbol{P}(2\omega)}{\partial t^2}$, where $\boldsymbol{P}(2\omega) = \varepsilon_0 \left(\chi^{(\mathrm{i})} + \chi^{(\mathrm{c})} \right) \colon \boldsymbol{E}(\omega)\boldsymbol{E}(\omega)$. The SHG signal measured experimentally is the intensity $I(2\omega)$ of the frequency-doubled light, and its relationship with $\boldsymbol{P}$ is: $I(2\omega) \propto \left| \boldsymbol{P}(2\omega) \right|^2$.

To analyze SHG experimental data, the second-order nonlinear optical susceptibility tensor should be represented in the laboratory coordinate system (x, y, z), as shown in the schematic diagram of Fig. 2A. Here, the incident angle θ is 45° and the azimuth angle α is 0°, so that the laboratory coordinate system and the crystal coordinate system are parallel. By rotating the polarization angle φ of the incident light and fixing the polarization of the reflection light as p ($\phi = 0°$) or s ($\phi = 90°$), the variation of the SHG intensity can be obtained. For non-centrosymmetric crystal point groups (such as the m point-group), the time-invariant SHG susceptibility tensor for the m point-group is in the form:

$$\chi^{(i)} = \begin{pmatrix} \chi_{11}^{(i)} & \chi_{12}^{(i)} & \chi_{13}^{(i)} & 0 & \chi_{15}^{(i)} & 0 \\ 0 & 0 & 0 & \chi_{24}^{(i)} & 0 & \chi_{26}^{(i)} \\ \chi_{31}^{(i)} & \chi_{32}^{(i)} & \chi_{33}^{(i)} & 0 & \chi_{35}^{(i)} & 0 \end{pmatrix}, \tag{1}$$

for p-out configuration, so ϕ = 0°. Substituting the time-invariant SHG susceptibility tensor $\chi^{(i)}$ into the $\boldsymbol{P}(2\omega)$, we have:

$$\boldsymbol{P}_{p-out}^{(i)}(2\omega) = Acos^2\varphi + Bsin^2\varphi + Csin2\varphi, \tag{2}$$

$$A = \frac{2\sqrt{2}L_{yy}^{2\omega}L_{yy}^{\omega}L_{zz}^{\omega}\chi_{24}^{(i)} - \sqrt{2}(L_{yy}^{\omega})^2 L_{zz}^{2\omega}\chi_{32}^{(i)} - \sqrt{2}(L_{zz}^{\omega})^2 L_{zz}^{2\omega}\chi_{33}^{(i)}}{4}, \tag{3}$$

$$B = -\frac{\sqrt{2}(L_{xx}^{\omega})^2 L_{zz}^{2\omega}\chi_{31}^{(i)}}{2}, \tag{4}$$

$$C = \frac{L_{yy}^{2\omega}L_{xx}^{\omega}L_{yy}^{\omega}\chi_{26}^{(i)} - L_{zz}^{2\omega}L_{xx}^{\omega}L_{zz}^{\omega}\chi_{35}^{(i)}}{2}, \tag{5}$$

in which L_{xx}, L_{yy}, and L_{zz} are the diagonal elements of the transmitted Fresnel factor L in fundamental and second harmonic optical materials. Because of $I(2\omega) \propto |\boldsymbol{P}(2\omega)|^2$,we obtained:

$$I_{p-out}^{(i)}(2\omega) \propto \left(Acos^2\varphi + Bsin^2\varphi + Csin2\varphi\right)^2. \tag{6}$$

Below the Néel temperature (about 618 K), the existence of the antiferromagnetic order in BFO films break the time-reversal symmetry. As the non-centrosymmetric magnetic order also break the spatial-inversion symmetry, the magnetization-induced ED-SHG is produced. With magnetic point-group m, the time-noninvariant SHG susceptibility tensor is [8]:

$$\chi^{(c)} = \begin{pmatrix} \chi_{11}^{(c)} & \chi_{12}^{(c)} & \chi_{13}^{(c)} & 0 & \chi_{15}^{(c)} & 0 \\ 0 & 0 & 0 & \chi_{24}^{(c)} & 0 & \chi_{12}^{(c)} \\ \chi_{15}^{(c)} & \chi_{24}^{(c)} & \chi_{33}^{(c)} & 0 & \chi_{13}^{(c)} & 0 \end{pmatrix}. \quad (7)$$

Similarly, substitute the time-noninvariant SHG susceptibility tensor $\chi^{(c)}$, we have:

$$\boldsymbol{P}_{p-out}^{(c)}\left(2\omega\right) = Dcos^2\varphi + Esin^2\varphi + Fsin2\varphi\,, \quad (8)$$

$$D = \frac{2\sqrt{2}L_{yy}^{2\omega}L_{yy}^{\omega}L_{zz}^{\omega}\chi_{24}^{(c)} - \sqrt{2}(L_{yy}^{\omega})^2 L_{zz}^{2\omega}\chi_{24}^{(c)} - \sqrt{2}(L_{zz}^{\omega})^2 L_{zz}^{2\omega}\chi_{33}^{(c)}}{4}\,, \quad (9)$$

$$E = -\frac{\sqrt{2}(L_{xx}^{\omega})^2 L_{zz}^{2\omega}\chi_{15}^{(c)}}{2}\,, \quad (10)$$

$$F = \frac{L_{yy}^{2\omega}L_{xx}^{\omega}L_{yy}^{\omega}\chi_{12}^{(c)} - L_{zz}^{2\omega}L_{xx}^{\omega}L_{zz}^{\omega}\chi_{13}^{(c)}}{2}\,. \quad (11)$$

Because of $I\left(2\omega\right) \propto \left|\boldsymbol{P}\left(2\omega\right)\right|^2$, we obtained:

$$I_{p-out}^{(c)}\left(2\omega\right) \propto \left(Dcos^2\varphi + Esin^2\varphi + Fsin2\varphi\right)^2. \quad (12)$$

References

1. Rana DS, Takahashi K, Mavani KR, Kawayama I, Murakami H, Tonouchi M, *et al.* Thickness dependence of the structure and magnetization of $BiFeO_3$ thin films on $(LaAlO_3)_{0.3}(Sr_2AlTaO_6)_{0.7}$ (001) substrate. *Physical Review B* 2007, **75**(6)**:** 060405.
2. Huang F, Lu X, Lin W, Kan Y, Zhang J, Chen Q, *et al.* Thickness-dependent structural and magnetic properties of $BiFeO_3$ films prepared by metal organic decomposition method. *Appl Phys Lett* 2010, **97**(22)**:** 222901.
3. Haykal A, Fischer J, Akhtar W, Chauleau JY, Sando D, Finco A, *et al.* Antiferromagnetic textures in $BiFeO_3$ controlled by strain and electric field. *Nature Communications* 2020, **11**(1)**:** 1704.
4. Zhang Y, Huang D, Shan Y, Jiang T, Zhang Z, Liu K, *et al.* Doping-Induced Second-Harmonic Generation in Centrosymmetric Graphene from Quadrupole Response. *Phys Rev Lett* 2019, **122**(4)**:** 047401.
5. Y.-R. Shen, *The Principles of Nonlinear Optics* (Wiley, New York, 1984).
6. Fiebig M, Fröhlich D, Krichevtsov BB, Pisarev RV. Second Harmonic Generation and Magnetic-Dipole-Electric-Dipole Interference in Antiferromagnetic Cr_2O_3. *Phys Rev Lett* 1994, **73**(15)**:** 2127-2130.
7. Birss RR. *Symmetry and magnetism*, vol. 3. North-Holland Publishing Company, 1964.
8. Chauleau JY, Haltz E, Carrétéro C, Fusil S, Viret M. Multi-stimuli manipulation of antiferromagnetic domains assessed by second-harmonic imaging. *Nat Mater* 2017, **16**(8)**:** 803-807.
